# Hyalinising clear cell carcinoma of the lung: A case report and review of literature

**DOI:** 10.1097/MD.0000000000034101

**Published:** 2023-06-23

**Authors:** Liqiao Chen, Ning Zhou, Shuya Hu, Fanrong Wang, Tingting Xu, Tao Li, Yangyan Fu, Yiman Luo, Ying Chen

**Affiliations:** a Department of Pathology, Sichuan Mianyang 404 Hospital, Mianyang, China; b Department of Pathology, Guiqian International General Hospital, Guiyang, China.

**Keywords:** clinicopathology, *EWSR1* rearrangement, Hyalinising clear cell carcinoma, lung neoplasm

## Abstract

Hyalinising clear cell carcinoma (HCCC) of the lung is a rare tumor, with only 12 reported cases. To improve the differential diagnosis, the aim of this study was to clarify the clinicopathological characteristics, immunophenotype, and molecular characteristics of HCCC of the lung and relate these to prognosis.

**Methods:**

Sections of HCCC of the lung were collected from a patient for pathological observation, immunohistochemistry, histochemistry, and fluorescence in situ hybridization; the clinical, pathological, and molecular characteristics were compared with those reported in the literature.

**Results:**

The tumor had a well-demarcated border nodule with a maximal diameter of 2.5 cm. Microscopic findings showed either clear or eosinophilic cytoplasm in the tumor cells. Growth was predominantly in the sheets, nests, and trabeculae in a background of hyalinised, fibrotic stroma, and mucus degeneration. Immunohistochemistry showed that the tumor cells expressed cytokeratin 7, P63, P40, CK5/6, Pan Cytokeratin (PCK), and epithelial membrane antigen, whereas they were negative for thyroid transcription factor-1, napsin A, CD10, vimentin, and smooth muscle actin. The Ki67 proliferation index was 5%. The tumor was positive for both period acid–Schiff (PAS) and Alcian blue–PAS, with a small amount of mucus staining positive for PAS–diastase. Fluorescence in situ hybridization revealed Ewing sarcoma breakpoint region 1 rearrangement and Ewing sarcoma breakpoint region 1-activating transcription factor 1 fusion.

**Conclusions:**

HCCC is a low-grade carcinoma with excellent prognosis. Tumour necrosis may be a potential risk factor for recurrence and metastasis. Our review of reported cases suggests that regional lymph node dissection combined with lobectomy is a safer treatment than only lobectomy for HCCC of the lung.

## 1. Introduction

Hyalinising clear cell carcinoma (HCCC) is a rare malignant tumor of salivary gland origin, often occurring in the minor salivary glands. This tumor was first described and named by Milchgrub et al^[1]^ in 1994. The upper jaw is the most frequently affected site, followed by the buccal mucosa, base of the tongue, floor of the mouth, lips, posterior molars, and tonsils. This tumor typically presents as a painless mass under the mucosa, with ulcers on the surface mucosa. The clinical symptoms vary depending on the location of the primary tumor.^[2]^

Primary lung salivary gland cancer is especially rare, accounting for less than 1% of all lung tumors. It originates from the small salivary glands under the bronchial mucosa.^[3]^ In the 2021 version of the World Health Organisation (WHO) classification of chest tumors, salivary gland lung epithelial tumors included pleomorphic adenoma, mucoepidermoid carcinoma, adenoid cystic carcinoma, epithelial-myoepithelial carcinoma, myoepithelioma, myoepithelial carcinoma, and HCCC. HCCC of the lung is the rarest salivary gland tumor, and it is mainly located in the central airway bronchus. It was first reported in 2015 by García et al^[4]^ and only 12 cases have been reported to date in global English-language literature.^[4–12]^

## 2. Materials and methods

### 2.1. Clinical sample

A nonsmoker, 54-year-old female patient was admitted to our hospital. A nodule in the anterior segment of the right upper lobe was incidentally identified via chest computed tomography (CT). The patient occasionally suffered from chest tightness but had no cough, sputum, hemoptysis, shortness of breath, or other discomforts. A chest CT scan revealed a single solid nodule of 24 × 15 mm located in the anterior segment of the right upper lobe; it presented lobulation signs, uneven enhancement, and reduced central density of the venous-phase lesion. Right upper lobectomy and regional lymph node dissection were performed, and no evidence of lymph node metastasis was found. The surgical margin was negative, and no further treatment was given.

### 2.2. Immunohistochemistry and histochemistry

The surgical specimen was fixed in 10% neutral buffered formalin, embedded in paraffin, and cut into 2.5 to 4 μm-thick sections. The sections were processed for hematoxylin and eosin staining, immunohistochemistry, and histochemistry. The DAKO automatic immunohistochemistry instrument (Agilent, Santa Clara, CA) was used. The antibodies and kits used were purchased from DAKO (Agilent) and Beijing Zhongshan Jinqiao Biotechnology Co., Ltd. (Beijing, China) and used according to the manufacturers’ instructions. The kits for histochemistry of periodic acid–Schiff (PAS), Alcian blue/PAS (AB–PAS), and PAS-diastase (D–PAS) were purchased from Guangzhou Anbiping Pharmaceutical Technology Co., Ltd. (Guangzhou, China) and were operated according to the manufacturer’s instructions.

### 2.3. Fluorescence in situ hybridization

Sections were first stained with hematoxylin and eosin to locate the tumor area. Then, to enable the observation of 100 tumor cells, the areas with clearly visible cell nuclei were selected under a fluorescent microscope. A red-green separation signal in the Ewing sarcoma breakpoint region 1 (*EWSR1*) gene indicates that the gene is broken. When >15% of the tumor cells showed this signal, it was judged as a positive gene rearrangement. Double staining of the *EWSR1* gene (green) and activating transcription factor 1 (*ATF1*) gene (red) was interpreted as gene fusion when >5% of tumor cells showed a positive signal; if the ratio of double fusion was below this threshold, single fusion in >30% of tumor cells was also interpreted as a positive signal for gene fusion. The probes and various reagents used in fluorescence in situ hybridization detection were purchased from Guangzhou Anbiping Pharmaceutical Technology Co., Ltd. (Guangzhou, China), and the specific operation steps were performed in strict accordance with the manufacturer’s instructions.

## 3. Results

### 3.1. Gross and microscopic findings

**Figure 1.**
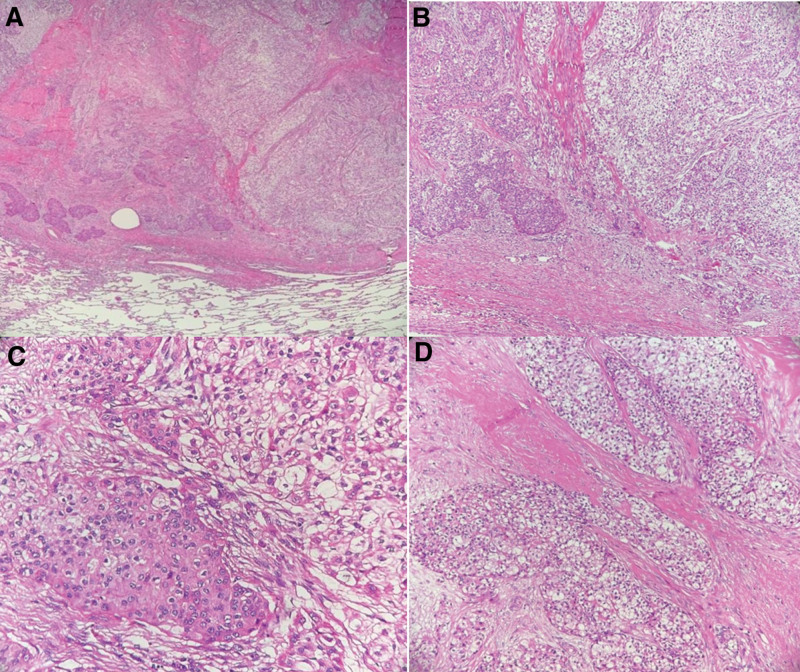
Histopathologic characteristics: (A) tumour detected in the lung; 2 mixed tissue components can be seen (hematoxylin and eosin, 4×). (B) Enlarged image of the right field of view of (A); the left side of the panel is dominated by eosinophils, and the right side is dominated by clear cells (hematoxylin and eosin, 10×). (C) Cytoplasm of tumor cells is transparent or eosinophilic and rich in cytoplasm (hematoxylin and eosin, 20×). (D) Cells are arranged in nestEs, with interstitial fibrosis and hyaline degeneration (hematoxylin and eosin, 20×).

A lung tissue section (with sutures and staples) was harvested from the patient with a volume of 10.5 × 10 × 2.5 cm^3^. The suture was dissected, revealing the nodule. The largest diameter was 2.5 cm, and the distance from the capsule was approximately 1.5 cm. The boundary was clear, and the nodule section was gray, solid, and medium in quality. Tumour cells were arranged in sheets, nests, and beams; the cells were epithelioid, eosinophilic, or transparent in the cytoplasm, characterized by interstitial fibrosis, hyalinosis, and mucus degeneration (Fig. 1).

### 3.2. Immunohistochemistry and histochemistry

**Figure 2.**
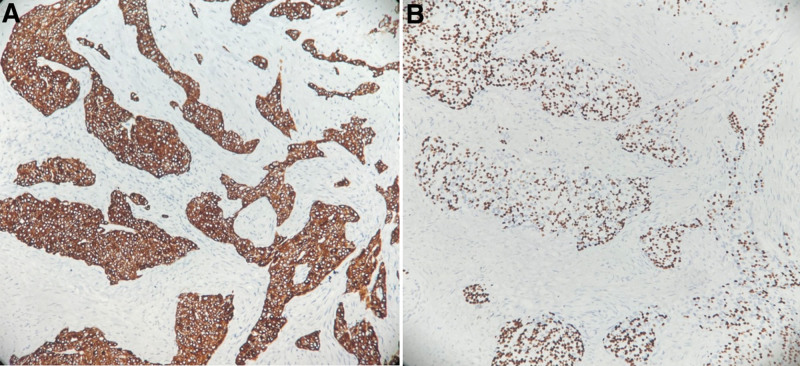
Immunohistochemistry: (A) tumour cells strongly positive for CK7 (20×). (B) Tumour cells strongly positive for P63 (×20).

Immunohistochemistry showed that the tumor cells were positive for CK7, P63 (Fig. 2), P40, CK5/6, and PCK; slightly positive for EMA; and negative for thyroid transcription factor-1 (TTF-1), napsin A, CD10, Vimentin, and smooth muscle actin. The Ki67 proliferation index was 5%. The tumor was also positive in AB-PAS and PAS staining, with a small amount of mucus staining positive for D-PAS.

### 3.3. *EWSR1* rearrangement and fusion

**Figure 3.**
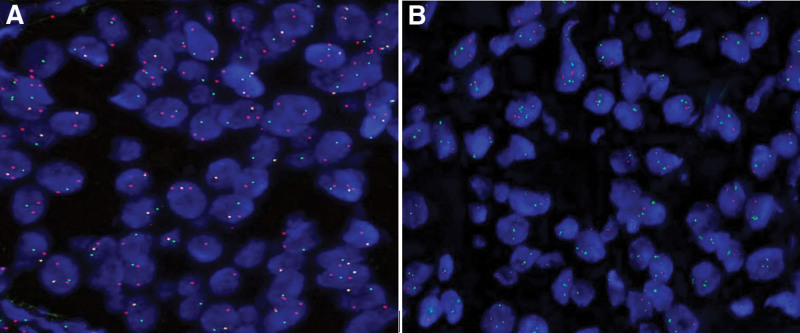
Molecular characteristics: (A) fluorescence in situ hybridization showing *EWSR1* rearrangement (100×). (B) Fluorescence in situ hybridization detection confirming *EWSR1–ATF1* fusion (100×).

Using the double-color separation break probe for *EWSR1*, the proportion of positive cells was 65%. This is above the 15% threshold; thus, the tissue demonstrated *EWSR1* gene rearrangement. The ratio of positivity for the double-color fusion probes for *EWSR*1 and *ATF1* was 7%, which was greater than the 5% threshold; the ratio of single fusion-positive cells was 71%, which was greater than 30%. These results, thus, confirmed the presence of *EWSR1–ATF1* gene fusion (Fig. 3).

## 4. Discussion

HCCC was included in the 2021version of the WHO lung tumor classification as an extremely rare entity, accounting for approximately 0.09% of all primary lung tumors.^[5]^ Only 12 cases have been reported worldwide to date. Given the rarity of HCCC of the lung, it is currently difficult to diagnose. Here, we describe a new case of HCCC of the lung, detected in a 54-year-old nonsmoker. We analyzed the clinicopathological characteristics, immunophenotype, and molecular genetic characteristics of the tumor sample and then compared these features with those reported for the other 12 cases of HCCC of the lung in the literature (Table 1, as additional files). Additionally, we analyzed the correlation between the clinicopathological characteristics and prognosis. These findings provide insight into the pathology of HCCC of the lung and will contribute towards improving the accuracy of diagnosis and deepening our understanding of the outcome of the disease. The results may also provide a reference for guiding clinical treatment.

**Table 1:** All reported cases of hyalinizing clear cell carcinoma of the lung.

Case	Age	Sex	Smoking	Site	Maximum tumor size	Imaging	Clinical stage and medical history	Histopathologic characteristics	Immunohistochemistry and histochemistry	Molecular characteristics	Treatment	Prognosis
1^[4]^	38	Male	Non-smoker	Right, lower lobe	2.6 cm	Solitary right perihilar mass; 3.03 cm in greatest dimension; in the vicinity of a large airway	No other pertinent medical history	Epithelioid tumor cells predominantly arranged in cords and occasional nests. Tumour cells growing in a background of hyaline fibrosis. Epithelioid appearance with eosinophilic or clear cytoplasm, open chromatin, and prominent nucleoli	Positive: PCK, CK7, P63, P40, and mucicarmine Negative: CK20, CgA, Syn, TTF-1, napsin A, S-100, and SMA	FISH: EWSR1 rearrangement was positive and MAML2 rearrangement was negative RT-PCR and Sanger sequencing: EWSR1-ATF1 fusion transcript	Right lower lobectomy	Alive and disease-free 10 mo after the diagnosis
2^[6]^	32	Male	Non-smoker	Left, lower lobe	1.8 cm	1.6 cm mass in left lower lobe	N/A	Infiltrative neoplasms comprising irregular nests, cords, and trabeculae of polygonal tumor cells with clear to eosinophilic cytoplasm. Tumour cells monotonous with irregular nuclear contours and inconspicuous nucleoli. Intranuclear cytoplasmic inclusions present. Tumour cellnests embedded in hyalinised stroma. Small foci of tumor necrosis present within nests	Positive: PCK, CK7, and P63 Negative: CK20, CD10, PAX-8, CgA, Syn, HMB-45, TTF-1, napsin A, S-100, and SMA	FISH: EWSR1 rearrangement	Lobectomy	Free of any local recurrence, metastasis after 1.5 yr of follow-up despite receiving no additional therapy after surgery
3^[6]^	39	Male	Non-smoker	Right, lower lobe	2.6 cm	2 cm mass in right lower lobe	N/A	Infiltrative neoplasms comprising irregular nests, cords, and trabeculae of polygonal tumor cells with clear to eosinophilic cytoplasm. Tumour cells monotonous with irregular nuclear contours and inconspicuous nucleoli. Tumour cellnests embedded in hyalinised stroma	Positive: PCK, CK7, and P63 Negative: CK20, CDlO, PAX-8, CgA, Syn, HMB-4, 1-TTF, napsin A, S-100, and SMA	FISH: EWSR1 rearrangement positive and MAML2 rearrangement negative	Right lower lobectomy	Free of any local recurrence. Metastasis after 1.5 yr of follow-up despite receiving no additional therapy after surgery
4^[8]^	54	Female	Ex-smoker (<one pack per week × 3 yr)	Left, upper lobe	3.5 cm	Interval enlargement of 3.2 cm left to suprahilar mass; slight interval enlargement of 7 mm noncalcified pulmonary nodule in lateral left lower lobe	pT2aN0, hypertension, gastroesophageal reflux disease, and intermittent chest pain	Tumour cell growth predominantly in sheets, nests, and cords in a background of hyalinised stroma. Tumour nuclei cytologically bland, nucleoli small or inconspicuous, and chromatin fine or vesicular. Rare intranuclear pseudo inclusions identified. No evidence of necrosis or nuclear pleomorphism. Mitotic figure count of an average one perten high-power fields	Positive: PCK, CK5/6, and P63 Negative: CK7, CK20, CgA, Syn, TTF-1, and napsin A	Next-generation sequencing: EWSR1-ATF1fusion	Left upper lobectomy with hilar and mediastinal lymphadenectomy	No evidence of recurrence or metastatic disease after 16 mo post-surgery
5^[7]^	53	Male	N/A	Right upper lobe in 1999; both lungs and enlarged group 3/4 lymph nodes in 2015	1.6 cm	N/A	N/A	Tumours comprised relatively small, polygonal epithelial cells with round nuclei, indistinct nucleoli, and eosinophilic or clear cytoplasm. Tumour cells formed an astomosing cords or solid nests with highly irregular borders in a fibrotic-to-hyalinised background. Glandular structures with mucin production confirmed with mucicarmine stain. No keratinization, tumor necrosis was observed	Positive: CK (AE1/AE3), mucicarmine, and PAS Negative: p40, p63, TTF-1, napsin A, Syn, CgA, S100, SMA, PAX8, SOX10, and calretinin	FISH: EWSR1 rearrangement	Right upper lobe lobectomy in 1999; Wedge resections and group 3/4 lymph node dissection through video-assisted thoracoscopic surgery in 2015	Metastasis after 16 yr
6^[9]^	55	Male	Smoking cessation 17 years earlier	Right, middle, and lower lobe	2.5 cm	Mass partially obstructing right bronchus intermedius	Chronic obstructive pulmonary disease and back pain	Nuclei appeared fairly monotonous with open chromatin and inconspicuous nucleoli. Neoplastic cells possessed either clear or pale eosinophilic cytoplasm. Mitotic activity minimal and necrosis not identified. Foci of peritumoral lymphocytic infiltration noted	Positive: CK7, CK5/6, CK34BE12, P63 and P40 Negative: TTF-1, napsin A, CK20, S100, SMA, Syn, and CgA	FISH: EWSR1 rearrangement	Video-assisted thoracoscopic bilobectomy (right middle and lower lobes) and mediastinal lymph node dissection	Recurrence-free 20 mo after diagnosis
7^[5]^	52	Female	nonsmoker	Right, segmental bronchus (lower lobe, between B9 and B10)	3.3 cm	N/A	N/A	Tumour cells had round to oval nuclei with inconspicuous nucleoli and clear to eosinophilic cytoplasm. Proportions of cells with clear or eosinophilic cytoplasm differed. Tumour cells grew with prominent trabecular pattern and nest formation	Positive: CK7, CK 5/6, p40, p63, Ki-67 (3–10%). Negative: TTF1, napsin A, HMB45, and Melan A	FISH: EWSR1 rearrangement;EWSR1-ATF1fusion RT-PCR (Sanger sequencing): EWSR1-ATF1 fusion	Right middle–lower lobectomy	Relapse-free survival of 181 mo
8^[5]^	35	Female	15 per day for 20 yr	Right, secondary bronchus (lower to middle lobar bronchus)	2.8 cm	Complete atelectasis of right lower lung (chest X-ray)	Persistent coughing with fever	Tumour cells had round to oval nuclei with inconspicuous nucleoli and clear to eosinophilic cytoplasm. Proportions of cells with clear or eosinophilic cytoplasm differed. Tumour cells grew with prominent trabecular pattern and nest formation	Positive: CK7, CK5/6, p40 p63, and Ki-67 (3–10%) Negative: TTF1, napsin A, HMB45, and Melan A	FISH: EWSR1 rearrangement;EWSR1-ATF1fusion RT-PCR (Sanger sequencing): EWSR1-ATF1 fusion	Right middle–lower lobectomy with regional lymph node dissection	Relapse-free survival 79 mo
9^[5]^	56	Female	Nonsmoker	Right, secondary bronchus (lower to inferior lobar bronchus)	3.3 cm	Abnormal shadow on chest X-ray	N/A	Tumour cells had round to oval nuclei with inconspicuous nucleoli and clear to eosinophilic cytoplasm. Proportions of cells with clear or eosinophilic cytoplasm differed. Tumour cells grew with prominent trabecular pattern and nest formation	Positive: CK7, CK5/6, p40, p63, and Ki-67 (3–10%) Negative: TTF1, napsin A, HMB45, and Melan A	FISH: EWSR1 rearrangement;EWSR1-ATF1fusion RT-PCR (Sanger sequencing): EWSR1-ATF1 fusion	Right middle–lower lobectomy with regional lymph node dissection	Relapse-free survival 12 mo
10^[12]^	55	Female	Smoker	Distal trachea	2.5 cm	2.5 cm intraluminal polypoid distal tracheal mass	Haemoptysis	Loose clusters and single cells with round, uniform nuclei, small prominent nucleoli, and abundant wispy light blue cytoplasm	Positive: CK7, p40 and CK5/6, p63 Negative: S-100 and SOX-10	FISH: EWSR1 rearrangement	Distal tracheal resection with mediastinal LN resection and adjuvant radiation therapy	No evidence of recurrent disease 12 mo after initial diagnosis
11^[10]^	75	Female	N/A	Lower lobe	0.9 cm	N/A	N/A	Infiltrative tumor with cells arranged in sheets, nests, cords, and trabeculae with intervening hyalinised eosinophilic stroma and lymphocytic infiltrate. Cells low-grade with variably clear to lightly eosinophilic cytoplasm and monomorphic basophilic nuclei	Positive: CK7, p63, CK14, and PAS-D Negative: S-100, SOX10, and DOG1	FISH: EWSR1 and CREM rearrangement EWSR1 exon 14 with CREM exon 6 fusion MAML2 break negative	Resection of the lower lobe of lung	No evidence of disease 8 mo post-surgery
12^[11]^	66	Female	Smoking cessation 6 yr previous (30 pack-years)	Trachea	1.3 cm	1.3 × 1.1 × 1.1-cmpolypoid mass arising from left posterior-lateral wall of trachea ~2 cm above carina	Hypertension, worsening shortness of breath	Nests of cells with monomorphic nuclei and clear cytoplasm set in a hyalinised stroma	Positive: p63, AE1/AE2, CK7, and CK5 Negative: TTF-1, napsinA, Syn, CgA, and S-100	FISH: EWSR1 rearrangement	Laser therapy and tumor debulking with cryotherapy to tumour base	N/A
The present case	54	Female	Nonsmoker	Right, upper lobe	2.5 cm	Single solid nodule (24 mm × 15 mm) in anterior segment of right upper lobe; signs of lobes, uneven enhancement, and reduced central density of venous lesions	Occasional chest tightness	Tumour cells arranged in sheets, nests, and beams; cells epithelioid, eosinophilic, or hyaline-like; interstitial fibrosis, hyaline degeneration, and mucus degeneration	Positive: p63, p40, CK7, CK5/6, PCK, EMA (minority +), Ki-67 (5%), AB-PAS, PAS, and D-PAS (small amount of mucus +) Negative: TTF-1, napsinA, SMA, CD10, and Vimentin	FISH: EWSR1 rearrangement; EWSR1-ATF1fusion	Right upper lobectomy with regional lymph node dissection	Recurrence- free 12 mo after diagnosis

ATF1 = activating transcription factor 1, CgA = chromogranin, EWSR1 = Ewing Sarcoma Breakpoint Region 1, FISH = fluorescence in situ hybridization, PAS = period acid–Schiff, PCK = Pan Cytokeratin, RT-PCR = reverse transcription polymerase chain reaction, SMA = smooth muscle actin, Syn = synaptophysin, TTF-1 = thyroid transcription factor-1.

Based on the reported literature and this case, the average age of patients with HCCC of the lung is 55 years, with a median of 54 years and a range of 32 to 75 years. The tumor appears to be more common in women, as 62% (8/13) of the reported cases were female patients. A slight majority (55%, 6/11) of the patients had no history of smoking, and none of the patients had a history of salivary gland tumors. Clinically, some patients had varying degrees of cough and shortness of breath, and a few patients had other symptoms such as intermittent chest pain, persistent back pain, hemoptysis, and dyspnea. CT or X-ray showed solid nodules in the lungs with clear nodules, some of which protruded into the bronchus, lung, or around the hilar. In 8 cases, the tumors were in the right lung; in 2 cases, they were in the left lung; and in the other 3 cases, they were in the trachea. The maximum tumor diameter ranged from 0.9 to 3.5 cm. The average tumor size was 2.4 cm, and the median maximum diameter was 2.5 cm. The tumor typically has a clear border, and the cut surface is white or off-white.

The histological morphology of HCCC of the lung appears to be common among the reported cases and the present case. The tumor cells are distributed in irregular nests, cords, and trabeculae, with round or oval nuclei. Most of the nucleoli are not obvious, the chromatin is fine, the nuclear atypia is small, and mitotic phases are rare. Pseudo-inclusion bodies in the nucleus are occasionally seen (2 of the 13 cases). The cytoplasm is transparent or eosinophilic, distributed in the hyaline or fibrotic stroma, and lymphocyte infiltration can be seen around and within the tumor. Occasionally, glands are formed, and mucin is secreted in the cavity. There is no formation of keratinized beads detected in any case. Regarding the immunophenotype, most cases of HCCC of the lung express epithelial markers such as PCK, CK7, CK5/6, P63, and P40, but are typically negative for TTF-1, napsin A, CgA, Syn, and S-100. The Ki-67 proliferation index is usually low at around 5%, as in the present case.

Recent studies have confirmed that 87% to 91% of HCCC cases have *EWSR1* gene rearrangement.^[13–15]^ *EWSR1–ATF1* fusion is the most common fusion form, which is also a characteristic of HCCC of the lung. *EWSR1* gene rearrangement was detected in all 13 reported cases (100%), and *EWSR1–ATF1* fusion was detected in 6 patients (46%), including the present case. The *EWSR1–CREM* fusion was also detected in 1 patient. However, detection of *EWSR1* and a related partner was not performed for six of the reported cases; *MAML2* genetic testing was performed for three of these cases, and no rearrangement was found.

HCCC is generally considered to be a low-grade tumor, which is indolent in most case reports. According to the reported literature, recurrence and lymph node metastasis after a follow-up of 8 to 181 months was found in only 1 case (8%),^[7]^ indicating that the overall prognosis is relatively good. When considering the prognostic factors of the disease, we found that in the relapsed case,^[7]^ the patient had been previously diagnosed with lung cancer (16 years prior to the HCCC of the lung diagnosis) and had a right upper lobectomy at that time. The chest CT scan performed 16 years later showed that the paratracheal lymph nodes were enlarged, with a diameter of 3.4 cm. Both lungs showed multiple nodules with a maximum diameter of 1.5 cm. Wedge resection and paratracheal lymph node dissection were performed using thoracoscopy. Microscopic review of the primary lung tumor slices excised 16 years previously showed that the tumor cells had similar histological characteristics, and both *EWSR1* rearrangement and *EWSR1–ATF1* gene fusion were detected using fluorescence in situ hybridization. This confirmed that the case was a primary HCCC of the lung with delayed lung metastasis after 16 years. More importantly, necrosis of the tumor cells was observed in the lung metastases and paratracheal lymph nodes. According to the reports of HCCC occurring in the head and neck,^[16,17]^ tumor necrosis is a potential risk factor for recurrence and metastasis. Therefore, we speculate that the potential risk factors for HCCC of the lung recurrence and metastasis may also be related to tumor necrosis.

Regarding the treatment of HCCC of the lung, six of the reported cases were mainly treated by local surgical resection, 5 cases received local surgical resection and regional lymph node dissection, 1 case received adjuvant radiotherapy at the same time as resection, and 1 case received laser therapy and tumor debulking with cryotherapy to the tumor base. In the case of recurrence, only local surgical resection had been performed 16 years prior. Therefore, we speculate that, for this type of tumor, lobectomy combined with selected regional lymph node dissection may be a safer treatment, especially if there is necrosis in the tumor with an increased histological grade. In the present case, both right upper lobectomy and regional lymph node dissection were performed, with no evidence of lymph node metastasis. As the surgical margin was negative, no further treatment was given. The current follow-up is 8 months, and there has been no recurrence or metastasis. However, long-term follow-up is needed to make any inferences regarding the effectiveness of our treatment strategy in this case.

The histological characteristics of HCCC of the lung may be difficult to distinguish from those of other tumors with clear cell characteristics, including mucoepidermoid carcinoma (MEC), squamous cell carcinoma, adenocarcinoma with clear cell characteristics, metastatic clear cell renal cell carcinoma, and perivascular epithelioidcell tumors (PEComa). Falk et al^[2]^ demonstrated that MEC, as the most common salivary gland tumor in the lung system, is similar to HCCC tumor cells in terms of immunohistochemistry, with both tumors showing positive staining for CK5/6, CK7, P63, and P40. Histologically, MEC tumor cells are usually mainly composed of mucous cells, epidermal cells (squamous cells), and intermediate cells; clear cells and eosinophils may also be present. Incontrast, HCCC components are simpler, mainly comprising clear cells in the hyaline stroma. In terms of molecular genetics, MEC is mainly associated with *MECT1–MAML2* fusion, whereas HCCC is mainly associated with a rearrangement of the *EWSR1* gene. The differential diagnosis with PEComa lies in the fact that tumor cells of PEComa are rich in glycogen, although its cytoplasmic transparency is similar to that of HCCC. However, PEComa has characteristic expression of the melanin markers HMB45 and/or melan A but does not express markers characteristic for HCCC such as CK and squamous epithelium-related markers (e.g., P63, P40, or CK5/6); HCCC generally does not express HMB45, which can help to distinguish it from PEComa.

Because HCCC usually expresses P63, P40, and CK5/6, another important differential diagnosis is squamous cell carcinoma. However, compared with typical squamous cell carcinoma, HCCC shows low-grade cytological characteristics, the cell atypia is not obvious, and mitoses are easily identified. HCCC also has a lower cell proliferation index and non-angularity than squamous cell carcinoma.

Other differential diagnoses include adenocarcinoma with clear cell characteristics and metastatic clear-cell renal cell carcinoma. For this distinction, it is important to consider the main clinical history and molecular features such as the presence of *EWSR1* rearrangement or *EWSR1–ATF1* fusion. In addition, compared with most lung adenocarcinomas, immunohistochemical features are the main distinguishing point for HCCC, which is negative for TTF-1 and napsin A, in contrast to lung adenocarcinomas.

## 5. Conclusions

In summary, we have reported another case of a very rare HCCC of the lung and summarized the cases reported to date. To our knowledge, this is the most complete literature review on HCCC of the lung. With a total of 13 cases now reported, our case helps to provide new context for this rare disease. HCCC of the lung has similar histological, immunophenotypic, and molecular characteristics to those of HCCC of the head and neck. Therefore, understanding key histomorphological features and the location of tumors is essential for the diagnosis of the disease, and the characteristic *EWSR1*gene rearrangement is also a strong indicator for auxiliary diagnosis. HCCC of the lung is an indolent salivary gland cancer with a good prognosis; however, the factors affecting the prognosis are uncertain. Tumour necrosis may be a potential risk factor for recurrence and metastasis. Owing to the limited information, more cases are needed to identify common and distinguishing features, which can help to improve differential diagnosis. Our review and case report suggest that regional lymph node dissection combined with lobectomy may be a safer treatment than only lobectomy for HCCC of the lung; however, it remains to be verified whether this treatment influences prognosis.

## Acknowledgements

We would like to thank Editage (www.editage.cn) for English language editing.

## Author contributions

**Conceptualization:** Liqiao Chen, Ning Zhou.

**Data curation:** Liqiao Chen, Fanrong Wang, Tingting Xu, Shuya Hu, Ying Chen.

**Methodology:** Tao Li, Yangyan Fu, Yiman Luo.

**Validation:** Liqiao Chen, Ning Zhou, Shuya Hu.

**Writing – original draft:** Liqiao Chen.

**Writing – review & editing:** Ning Zhou.
